# Impact of age on the prognostic benefit of follow-up in UMIPIC units: analysis of the RICA registry

**DOI:** 10.1007/s11739-026-04371-5

**Published:** 2026-06-03

**Authors:** Pau Llàcer, François Croset, Álvaro González Franco, José Manuel Cerqueiro, Manuel Méndez, Manuel Montero-Pérez-Barquero, José Pérez Silvestre, Alicia Conde, Melitón Dávila, Margarita Carrera, Fernando Aguilar, Eduardo Martínez Litago, Francesc Formiga, Luis Manzano

**Affiliations:** 1 Internal Medicine Department, Hospital Universitario Ramón y Cajal, IRYCIS, Madrid, Spain; 2 Department of Medicine and Medical Specialties, Facultad de Medicina y Ciencias de la Salud, Universidad de Alcalá, IRYCIS, Madrid, Spain; 3 Internal Medicine Department, Hospital Universitario Central de Asturias, Oviedo, Spain; 4 Internal Medicine Department, Hospital Universitario Lucus Augusti, Lugo, Spain; 5 Internal Medicine Department, Hospital Clínico San Carlos, Instituto de Investigación Sanitaria del Hospital Clinico San Carlos (IdISSC), Madrid, Spain; 6 Faculty of Medicine, Universidad Complutense, Madrid, Spain; 7 Internal Medicine Department, IMIBIC, University Hospital Reina Sofía, Córdoba, Spain; 8 Internal Medicine Department, Consorcio Hospital General Universitario de Valencia, Valencia, Spain; 9 Internal Medicine Department, Hospital Universitario de Gran Canaria Dr. Negrín, Las Palmas de Gran Canaria, Las Palmas, Spain; 10 Health Sciences Faculty, Universidad de Las Palmas de Gran Canaria, Las Palmas, Spain; 11 Internal Medicine Department, Hospital Nuestra señora de la candelaria, Santa Cruz de Tenerife, Spain; 12 Internal Medicine Department, Complejo Hospitalario de Soria, Soria, Spain; 13 Internal Medicine Department, Hospital Universitario 12 de Octubre, Madrid, Spain; 14 Internal Medicine Department, Hospital Santa Bárbara de Puertollano, Ciudad Real, Spain; 15 Internal Medicine Department, Hospital Universitari de Bellvitge, Barcelona, Spain

**Keywords:** Acute heart failure, UMIPIC, Age, Interaction, Prognosis

## Abstract

Multidisciplinary management programs are recommended for patients with heart failure (HF), yet it remains uncertain whether their prognostic benefits extend uniformly across all ages, particularly among the very elderly. This study evaluated whether age modifies the benefit of follow-up in UMIPIC (Comprehensive Management Unit for Patients with Heart Failure), using data from the Spanish RICA registry. We conducted a prospective, multicenter cohort study including 5644 patients (mean age 79.9±8.6 years; 52.7% women) hospitalized for acute HF and followed for one year. Patients were managed either within UMIPIC units or with conventional care. The primary outcomes were all-cause mortality and HF readmissions. Cox regression models tested the interaction between UMIPIC and age, modeled categorically (<90 vs ≥90 years), continuously, and with restricted cubic splines. During a median follow-up of 363 days, 1.419 patients (25.1%) died and 1.287 (22.8%) were rehospitalized for HF. In multivariable Cox models, UMIPIC participation was independently associated with lower mortality, with a significant age interaction (p=0.026). Among patients, 90 years, UMIPIC follow-up reduced mortality by 40% (HR 0.60; 95% CI 0.50–0.73; p<0.001), with consistent benefit across age groups (p for interaction=0.971). UMIPIC follow-up was associated with lower mortality and rehospitalization in older adults with HF. The survival benefit was most pronounced among nonagenarians, supporting the inclusion of very old patients in multidisciplinary HF programs.

## Introduction

Heart failure (HF) is a common condition, affecting approximately 2% of the general population, with prevalence rising to nearly 15% among individuals over 80 years of age [1–4], and older adults frequently present with a high burden of both cardiac and non-cardiac comorbidities [1–6]. Moreover, heart failure rehospitalization and mortality increase with age. Contemporary evidence from large cohorts and meta-analyses indicates that rehospitalization after heart failure hospitalization remains frequent, with rates of 13–22% at 30 days and 30–61% at 1 year [6, 7]. These rates are higher among older patients and those with comorbidities. Mortality is also substantial and rises with age, reaching 20–37% at 1 year and 50–60% at 5 years in older adults [6, 7]. The high rates of rehospitalization constitute a substantial economic burden for the healthcare systems [7–9].

Clinical practice guidelines for HF, with the highest class and level of evidence, recommend multidisciplinary HF management programs as they reduce hospital admissions and mortality [10, 11]. Their incorporation into routine care is considered a cornerstone of contemporary heart failure management [10, 11]. Along these lines, the UMIPIC program (Comprehensive Management Unit for Patients with Heart Failure), developed by the Heart Failure Group of the Spanish Society of Internal Medicine (SEMI), has consistently demonstrated clinical benefits in patients with multimorbid HF [12, 13]. Its value lies in providing integrated, continuous care centered on the versatility and holistic approach of the internist.

However, it remains unclear whether the prognostic benefit of the UMIPIC program is homogeneous across all age groups. Older patients represent a particularly heterogeneous population, with wide variability in comorbidities, frailty, and functional reserve, factors that may modify both prognosis and the response to multidisciplinary care interventions. Identifying whether age influences the magnitude of UMIPIC benefit is crucial, as it could help target those subgroups most likely to achieve significant prognostic improvement.

Accordingly, the objective of this study was to evaluate whether age modifies the prognostic benefit of follow-up in UMIPIC units, using data from the RICA registry.

## Material and methods

### Study population

This prospective, multicenter, observational cohort study included patients with a diagnosis of HF who were either managed through the UMIPIC multidisciplinary program or received conventional care, as documented in the RICA (National Heart Failure Registry) database [12]. Since March 2008, the RICA Registry has prospectively, and consecutively enrolled patients admitted for acute heart failure (AHF) in the internal medicine departments of public and private hospitals across Spain. The design and methodology of the registry have been described in detail elsewhere [12].

Briefly, patients older than 50 years admitted for AHF who meet the diagnostic criteria of the European Society of Cardiology (ESC) [9] are prospectively included, regardless of clinical presentation, etiology, or left ventricular ejection fraction (LVEF). After the index admission, follow-up is performed with two mandatory visits, scheduled at 3 and 12 months (in addition to any other visits deemed necessary by the investigator according to clinical judgment).

The RICA registry includes monitoring procedures responsible for ensuring the quality control of the data entered. A patient is considered valid once follow-up is completed and the data has passed quality review by the monitor.

The type of post-discharge follow-up was non-randomized and determined by routine clinical practice. All patients were followed for a minimum of one year. Enrollment in the UMIPIC program depended on whether the hospital had such a unit available. The UMIPIC program characterized for patients enrolled in the Comprehensive Management Unit for Patients with Heart Failure (UMIPIC), a protocol-driven, multidisciplinary outpatient program specifically designed for older patients with chronic HF and multiple comorbidities. Care is delivered by internists and specialized nurses and is structured around five core components [12]: i) comprehensive management of HF and comorbidities, ii) continuous follow-up through in-person and telephone visits, iii) structured education of patients and caregivers to promote adherence, self-care, and early symptom recognition, iv) rapid access to medical attention for acute decompensations, and v) coordination with other specialists when needed. Care is individualized and consistent with guideline recommendations, including pharmacological optimization, lifestyle counseling, and functional monitoring. Enrollment in UMIPIC was reserved for patients at high risk of early readmission, identified by recurrent hospitalizations or emergency visits in the previous year, poor clinical status at discharge (such as renal dysfunction or high diuretic requirements), or the need for drug titration. A minimum level of cognitive and functional capacity, or the presence of a caregiver, was required to ensure adherence to the intensive follow-up protocol. On the other hand, conventional care (non-UMIPIC), the patients managed under usual care without a structured HF program.

### Study variables

At baseline, data were collected on sociodemographic variables, comorbidities (Charlson comorbidity index), functional status (Barthel index), LVEF assessed by echocardiography, laboratory parameters, and therapeutic data. Biochemical variables were obtained from the emergency laboratory tests performed at the index admission.

### Endpoints of interest

The primary outcomes were all-cause mortality and heart failure readmissions within one year after discharge.

### Statistical analysis

Continuous variables are presented as mean ± SD or median (percentile 25% to percentile 75%), and categorical variables are expressed as percentages. Baseline characteristics across age groups, categorized in < 90 years and ≥ 90 years, were compared using the χ^2^ (chi-square) test for categorical variables. For continuous variables, Student’s t-test for continuous variables was used if the data followed a normal distribution or the Kruskal–Wallis if the data were not normally distributed. A variable with 4 categories was created by combining age (< 90 vs. ≥ 90 years) and follow-up type (UMIPIC vs. non-UMIPIC): C1 =  < 90 years and non-UMIPIC; C2 =  < 90 years and UMIPIC; C3 =  ≥ 90 years and non-UMIPIC; C4 =  ≥ 90 years and UMIPIC.

Survival analysis was performed using Kaplan–Meier curves with log-rank tests to compare UMIPIC versus non-UMIPIC, stratified by age group. The statistical significance of the interaction between the care model (UMIPIC vs no UMIPIC) and both continuous and dichotomic age values was assessed for all endpoints. They included restricted cubic splines applied to age (without assuming a linear trend) with 3 knots located at the 10th, 50th, and 90th per centiles in case of a significant nonlinear association with age. Multivariable Cox proportional hazards regression models were fitted. In the multivariable model, all variables listed in Table 1 were tested based on prior knowledge/biological plausibility, regardless of the p- value. We simultaneously tested the linearity assumption for all continuous variables, and the variables were transformed using fractional polynomials when appropriate. Next, we derived a reduced and parsimonious model using backward stepwise selection on prior knowledge/biological plausibility, independent of the p-value. The covariates included in the final multivariable models were sex, hemoglobin, plasma sodium, estimate glomerular filtration rate (eGFR), systolic blood pressure, heart rate, LVEF, Charlson index, nursing home resident, betablockers, ACE/ARB/ARNI and interaction UMIPIC#age. Model discrimination was evaluated using Harrell’s C-statistic, which was 0.696 for all-cause mortality and 0.600 for rehospitalization. The corresponding AUC values were 0.704 and 0.595, respectively. To assess the robustness of our findings, we performed a propensity score analysis. The results were consistent and closely aligned with our primary analyses.

**Table 1:** Baseline characteristics across age groups

Variables	Total (n = 5644)	Age ≥ 90 years (n = 606)	Age < 90 years (n = 5038)	*p-value*
Demographics and medical history				
Age (years), mean (SD)	79.9 (8.6)	92.7(2.4)	78.9(8.1)	< 0.001
Female sex, n(%)	2974 (52.7)	342 (56.4)	2632 (52.2)	0.051
UMIPIC	2034 (36)	257 (42.4)	1777 (35.3)	0.001
Nursing home resident, N (%)	462 (8.2)	34 (5.6)	428(8.5)	0.014
Hypertension, N (%)	4866(86.2)	535(88.3)	4331(86)	0.118
Diabetes mellitus type 2, N (%)	2606 (46.2)	217(35.8)	2389(47.4)	< 0.001
Dyslipidemia, N (%)	2914(51.6)	292(48.2)	2622(52)	0.072
Obesity (BMI > 30), N (%)	2116 (37.5)	160 (26.4)	1956(38.8)	< 0.001
Atrial fibrillation, N (%)	3005 (53.2)	334 (55.1)	2671 (53)	0.328
Ischemic heart disease, N (%)	1452 (25.7)	151 (24.9)	1301 (25.8)	0.630
Chronic kidney disease, N (%)	3490 (61.8)	366 (60.4)	3124 (62)	0.440
Stroke, N (%)	786 (13.9)	81 (13.4)	705 (14)	0.673
Peripheral arterial disease, N (%)	619 (11)	47 (7.8)	572 (11.3)	0.007
Dementia, N (%)	307 (5.4)	39(6.4)	268 (5.3)	0.252
COPD, N (%)	1320 (23.4)	110 (18.2)	1210 (24)	0.001
Neoplasm, N (%)	668 (11.8)	75 (12.4)	593 (11.8)	0.663
Clinical Characteristics				
SBP (mmHg), mean (SD)	137.1 (26.5)	134.4(23.9)	137.5(26.7)	0.006
DBP (mmHg), mean (SD)	74.8(15.7)	72(13.8)	75.2(15.9)	< 0.001
HR (lpm), mean (SD)	86.4 (22.5)	82.1 (22)	86.9 (22.6)	< 0.001
Charlson Index, mean (SD)	3 (2.5)	2.7 (2.4)	3.1 (2.5)	< 0.001
Barthel Index (points), mean (SD)	82.7 (22.4)	76.8 (25.7)	83.5 (22)	< 0.001
Pfeiffer Test, mean (SD)	1.6 (2.1)	2 (2.3)	1.6 (2.1)	< 0.001
Characteristics of cardiopathy				
LVEF, mean (SD)	51.4 (15.7)	52.5(15.9)	51.2 (15.7)	0.064
LVEF > 50%, N (%)	3158 (56)	357 (57)	2801 (55.6)	0.121
NYHA				0.526
I	423 (7.5)	43 (7.1)	380 (7.5)	
II	3025 (53.6)	320 (52.8)	2705 (53.7)	
III	1915 (33.9)	208 (34.3)	1707 (33.9)	
IV	166 (2.9)	17 (2.8)	149 (3)	
Etiology of HF				
Hypertensive	2186 (38.7)	224 (37)	1962 (39)	0.344
Ischemic	1452 (25.7)	151 (24.9)	1301 (25.8)	0.630
Valvular	926 (16.4)	90 (14.9)	836 (16.6)	0.274
Unaffiliated	545 (9.7)	84 (13.9)	461 (9.2)	< 0.001
Laboratory				
Hemoglobin (g/dL), mean (SD)	12.1 (2)	12.1 (2)	12.1 (2)	0.641
Creatinine (mg/dL), mean (SD)	1.3 (1.7)	1.5 (4.9)	1.3(0.7)	0.010
eGFR, mean (SD)	58.9 (27.7)	57.2 (27.7)	59.1 (26.6)	0.100
NT- proBNP (pg/mL), median [IQR]	3374 (1620.5–7609.5)	3230 (1510–7969)	3394 (1632–7540)	0.625
Sodium (mEq/L), mean (SD)	138.7 (5.4)	139 (5.1)	138.7 (5.4)	0.252
Potassium (mEq/L), mean (SD)	4.3 (0.6)	4.4 (0.6)	4.3 (0.6)	< 0.001
Treatment				
Beta blockers, N (%)	3867 (68.5)	401 (66.2)	3466 (68.8)	0.189
ACE inhibitors/ARB/ARNI, N (%)	3659 (64.8)	369 (60.9)	3290 (65.3)	0.032
Aldosterone antagonists, N (%)	1303 (23.1)	124 (20.5)	1179 (23.4)	0.105
Loop diuretics, N (%)	4205 (74.5)	420 (69.3)	3785 (75.1)	0.002
Thiazide diuretics, N (%)	871 (15.4)	93 (15.4)	788 (15.4)	0.951
Digoxin, N (%)	1183 (21)	98 (16.2)	1085 (21.5)	0.002
Statins, N (%)	1707 (30.2)	180 (29.7)	1527 (30.3)	0.759
Events				
Heart Failure hospitalizations, n(%)	1287 (22.8)	116 (19.1)	1171 (23.2)	0.023
All-cause mortality, n(%)	1419 (25)	157 (25.9)	1262 (25)	0.646

We excluded NT-proBNP from the primary Cox regression models to minimize loss of patients due to missing values. As the results did not materially change when NT-proBNP was included, we present the models without this variable to preserve sample size and statistical power. To assess the robustness of our findings, we performed a sensitivity analysis including NT-pro BNP in the models. The overall discriminative ability of the models with NT-proBNP, assessed by Harrell’s C-statistic, was 0.692 for mortality (AUC 0.704) and 0.599 for rehospitalization (AUC 0.593).

## Results

### Baseline characteristics

A total of 5644 patients were included. The mean (SD) age was 79.9 ± 8.6 years, 52.7% were women, 3158 (56%) had HFpEF, and 2034 (36%) were enrolled in the UMIPIC program. The most prevalent comorbidities were hypertension (86.2%), atrial fibrillation (60%), chronic kidney disease (61.8%), diabetes mellitus (46.2%), obesity (37.5%), ischemic heart disease (25.7%), and COPD (23.4%).

### Baseline profiles across age groups

Enrollment in UMIPIC was more frequent among patients aged ≥ 90 years. Compared with younger patients, nonagenarians were less nursing home residents and had lower prevalence of diabetes, obesity, COPD, and peripheral arterial disease, while the prevalence of other major comorbidities was similar between groups (Table 1).

At admission, patients aged ≥ 90 years had lower systolic and diastolic blood pressures and a lower heart rate. They also showed a lower Charlson comorbidity index but worse functional and cognitive status, as reflected by lower Barthel index scores and higher Pfeiffer test scores. Left ventricular ejection fraction was similar between groups, with no significant differences in NYHA functional class distribution, and hypertensive etiology predominated in both age strata.

Laboratory findings were broadly comparable across age groups, with no significant differences in hemoglobin, renal function, or NT-proBNP concentrations.

Regarding treatment, the use of beta-blockers was high overall and similar between groups. Patients ≥ 90 years were less frequently prescribed ACE inhibitors/ARB/ARNI, loop diuretics, and digoxin. In contrast, the use of mineralocorticoid receptor antagonists, thiazide diuretics, and statins did not differ significantly between age groups.

When the cohort was analyzed according to the four categories, the baseline profile of patients aged ≥ 90 years did not substantially differ between those enrolled in UMIPIC and those not enrolled, except that C3 patients (≥ 90/non-UMIPIC) had a lower Charlson comorbidity index and a higher Barthel index, but worse cognitive status on the Pfeiffer test and a lower LVEF compared with C4 patients (≥ 90/UMIPIC) (Table 2).

**Table 2:** Baseline characteristics across age and UMIPIC categories

Variables	Total (n = 5644)	C1 <90 years/non-UMIPIC (n=3261)	C2 <90 years/UMIPIC (n=1777)	C3 ≥90 years/non-UMIPIC (n=349)	C4 ≥90 years/UMIPIC (n=257)	*p-value*
Demographics and medical history						
Age (years), mean (SD)	79.9(8.6)	77.7 (8.6)	81.1(6.7)	92.7 (2.7)	92.7 (2.2)	<0.001
Female sex, n (%)	2.974 (52.7)	1.667 (51.1)	965 (54.31)	189 (54.15)	153 (59.5)	0.017
Nursing home resident, N (%)	462 (8.2)	332 (10.2)	96 (5.4)	20 (5.7)	14 (5.5)	<0.001
Hypertension, N (%)	4.866 (86.2)	2750 (84.3)	1581 (89)	299 (85.7)	236 (91.8)	<0.001
Diabetes mellitus type 2, N (%)	2602 (46.2)	1542 (47.3)	847 (47.7)	120 (34.4)	97 (37.7)	<0.001
Dyslipidemia, N (%)	2914(51.6)	1.652 (50.7)	970 (54.6)	178 (51)	114 (44.4)	0.005
Obesity (BMI > 30), N (%)	2116 (37.5)	1258 (38.6)	698 (38.3)	90 (25.8)	70 (27.2)	<0.001
Atrial fibrillation, N (%)	3005 (53.2)	1655 (55.1)	1006 (56.6)	198 (56.7)	136 (52.9)	<0.001
Ischemic heart disease, N (%)	1452 (25.7)	895 (27.5)	406 (22.9)	102 (29.2)	49 (19.1)	<0.001
Chronic kidney disease, N (%)	3490 (61.8)	2165 (66.4)	959 (54)	214 (61.3)	152 (59.1)	<0.001
Stroke, N (%)	786 (13.9)	442 (13.6)	263 (14.8)	47 (13.5)	34 (13.2)	0.643
Peripheral arterial disease, N (%)	619 (11)	372 (11.4)	200 (11.3)	24 (6.9)	23 (9)	0.049
Dementia, N (%)	307 (5.4)	182 (5.6)	86 (4.8)	28 (8)	11 (4.3)	0.087
COPD, N (%)	1320 (23.4)	819 (25.1)	391 (22)	66 (18.9)	44 (17.1)	0.001
Neoplasm, N (%)	668 (11.8)	358 (11)	235 (13.2)	31 (8.9)	44 (17.1)	0.002
Clinical Characteristics						
SBP (mmHg), mean (SD)	137.1 (26.5)	138.9 (27.7)	134.8 (24.8)	134.3 (24.4)	134.5 (23.3)	<0.001
DBP (mmHg), mean (SD)	74.8(15.7)	76.4 (16.2)	73 (14.9)	72.7 (14.1)	71.1 (13.3)	<0.001
HR (lpm), mean (SD)	86.4 (22.5)	88.5 (23.2)	83.9 (21)	82.4 (22.2)	81.7 (20.6)	<0.001
Charlson Index, mean (SD)	3 (2.5)	3 (2.5)	3.3 (2.5)	2.6 (2.2)	3 (2.6)	0.023
Barthel Index (points), mean (SD)	82.7 (22.4)	84.5 (21.7)	81.3 (22.6)	79.1 (24.7)	72.9 (27)	<0.001
Pfeiffer Test, mean (SD)	1.6 (2.1)	1.7 (2.2)	1.4 (1.9)	2.1 (2.4)	1.8 (2.1)	<0.001
Characteristics of cardiopathy						
LVEF, mean (SD)	51.4 (15.7)	50.4 (16.1)	52.8 (14.8)	50.4 (16)	55.3 (15.4)	0.001
LVEF > 50%, N (%)	3158 (56)	1741 (53.4)	1060 (59.7)	194 (55.6)	163 (63.4)	<0.001
NYHA						<0.001
I	423 (7.5)	298 (9.1)	82 (4.6)	29 (8.3)	14 (5.5)	
II	3025 (53.6)	1712 (52.5)	993 (55.9)	177 (50.7)	143 (55.6)	
III	1915 (33.9)	1068 (32.8)	639 (36)	116 (33.2)	92 (35.8)	
IV	166 (2.9)	94 (2.9)	55 (3.1)	11 (3.2)	6 (2.3)	
Etiology of HF						
Hypertensive	2186 (38.7)	1148 (25.2)	814 (45.8)	95 (27.2)	129 (50.2)	<0.001
Ischemic	1452 (25.7)	895 (27.5)	406 (22.9)	102 (29.2)	49 (19.1)	<0.001
Valvular	926 (16.4)	577 (17.7)	259 (14.6)	55 (15.8)	35 (13.6)	0.020
Unaffiliated	545 (9.7)	344 (10.6)	117 (6.6)	56 (16.1)	28 (10.9)	<0.001
Laboratory						
Hemoglobin (g/dL), mean (SD)	12.1 (2)	12.1 (2.1)	11.9 (2)	12.3 (2)	11.8 (1.9)	0.015
Creatinine (mg/dL), mean (SD)	1.3 (1.7)	1.3 (0.7)	1.3 (0.7)	1.3 (0.7)	1.7 (7.5)	<0.001
eGFR, mean (SD)	58.9 (27.7)	59.3 (26.6)	58.8 (26.6)	57.5 (29)	56.8 (25.8)	0.115
NT- proBNP (pg/mL), median [IQR]	3374 (1620.5-7609.5)	3381 (1561-7341)	3408 (1850-7915)	3315 (1585-7720)	2900 (1348-8412)	0.346
Sodium (mEq/L), mean (SD)	138.7 (5.4)	138.8 (5.4)	138.5 (5.4)	139.4 (5)	138.4 (5.3)	0.191
Potassium (mEq/L), mean (SD)	4.3 (0.6)	4.3 (0.6)	4.4 (0.6)	4.5 (0.6)	4.4 (0.6)	0.568
Treatment						
Beta blockers, N (%)	3867 (68.5)	2606 (67.7)	1260 (60.9)	237 (67.9)	164 (67.8)	0.036
ACE inhibitors/ARB/ARNI, N (%)	3659 (64.8)	2205 (67.6)	1085 (61.1)	214 (61.3)	155 (60.3)	<0.001
Aldosterone antagonists, N (%)	1303 (23.1)	709 (21.7)	470 (26.4)	76 (21.8)	48 (18.7)	<0.001
Loop diuretics, N (%)	4205 (74.5)	2413 (74)	1372 (77.2)	231 (66.2)	189 (73.5)	<0.001
Thiazide diuretics, N (%)	871 (15.4)	424 (13)	354 (20)	41 (11.8)	52 (20.2)	<0.001
Digoxin, N (%)	1183 (21)	740 (22.7)	345 (19.4)	64 (18.3)	34 (13.2)	<0.001
Statins, N (%)	1707 (30.2)	934 (28.6)	593 (33.4)	117 (33.5)	63 (24.5)	<0.001
Events						
Heart Failure hospitalizations, n (%)	1287 (22.8)	1345 (45.3)	640 (36)	102 (29.2)	75 (29.2)	<0.001
All-cause mortality, n (%)	1419 (25)	881 (27)	381 (21.4)	102 (29.2)	55 (21.4)	<0.001

### Effect of age groups on the care model (UMIPIC/no UMIPIC) for the endpoints

#### All-cause mortality

**Fig. 1:**
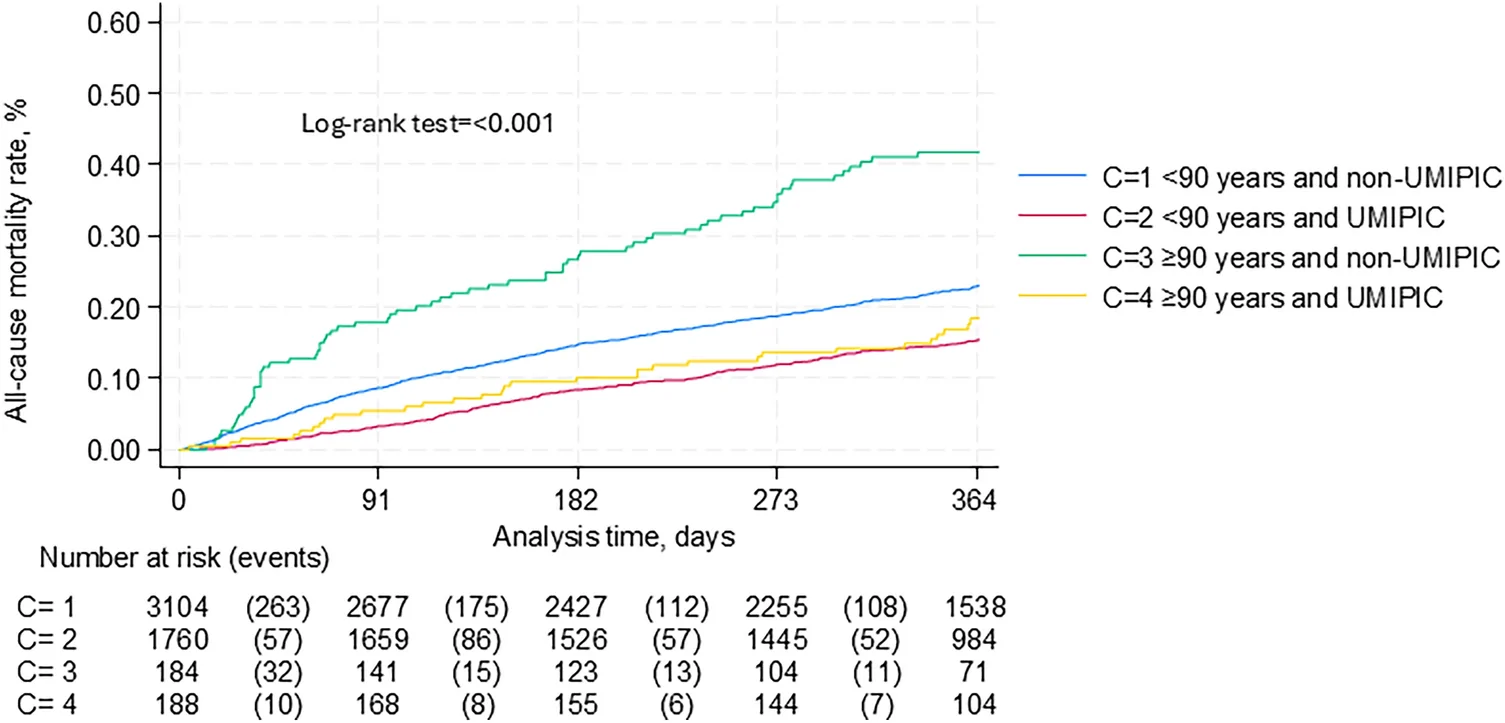
Kaplan-Meier curves age/UMIPIC categories for all-cause mortality

During a median (IQR, p25–p75) follow-up of 363 (236–391) days, 1419 (25.1%) patients died. Patients with ≥ 90 years presented similar rates of the 1-year all-cause mortality with < 90 years (25.9% vs 25%, p = 0.646). When stratifying the predictive value of the care model (UMIPIC vs. non-UMIPIC) by age, risk was reclassified differently across groups (C1 27%, C2 21.4%, C3 29.2%, C4 21.4%; p < 0.001). Kaplan–Meier curves demonstrated divergent trajectories, with the highest risk observed in patients ≥ 90 years managed outside UMIPIC, while no clear differences were noted among the other groups (log-rank test =  < 0.001) (Fig. 1). After multivariable adjustment, the association between care model (UMIPIC vs no UMIPIC) and outcomes continued to differ significantly by group age (p value for interaction = 0.026). When analyzing the interaction between age group and participation in the UMIPIC program, patients aged ≥ 90 years showed a hazard ratio (HR) of 0.30 (95% CI 0.17–0.54; p < 0.001), while those aged < 90 years had a HR of 0.60 (95% CI 0.50–0.73; p < 0.001), indicating that the program was protective in both groups, with an even greater relative benefit among the oldest patients.

Compared to the lower baseline risk group (reference category: patients < 90 years enrolled in UMIPIC, C = 2), patients < 90 years without UMIPIC follow-up (C = 1) had a 61% higher risk of death (HR 1.66, 95% CI 1.38–1.96; p < 0.001). The highest risk was observed in patients ≥ 90 years without UMIPIC (C = 3), with a more than threefold increase in mortality (HR 3.29, 95% CI 2.40–4.52; p < 0.001). In contrast, patients ≥ 90 years enrolled in UMIPIC (C = 4) had no significant excess risk compared with the reference group (HR 0.99, 95% CI 0.859–1.70; p = 1.000). When analyzing age as a continuous variable, the association with adverse events differed by care model (UMIPIC vs. non-UMIPIC), showing a stronger association between increasing age and risk among patients managed outside UMIPIC (p for interaction = 0.048), indicating that the adverse impact of increasing age was attenuated among patients enrolled in UMIPIC compared with those receiving usual care.

**Fig. 2:**
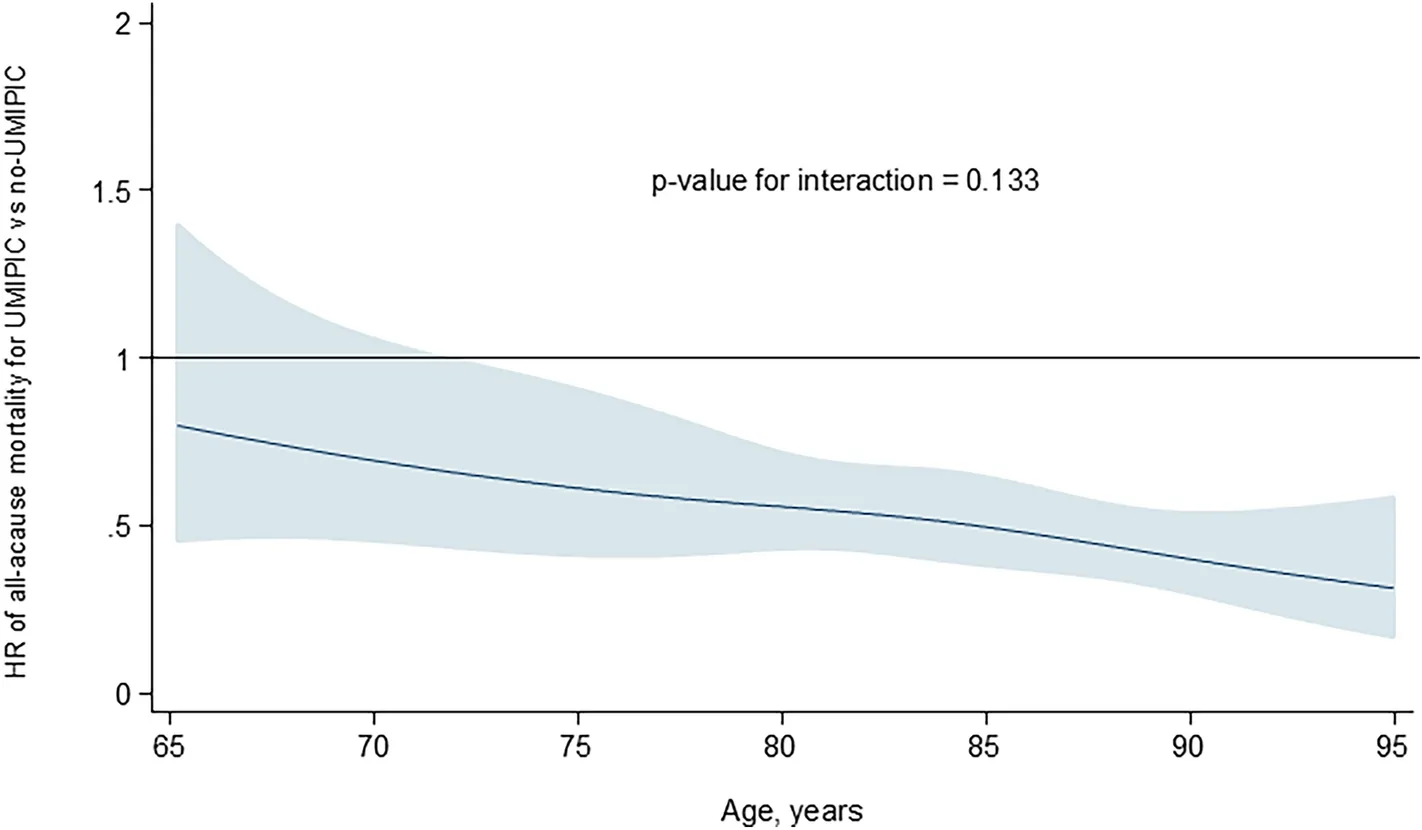
Effect of age on the association between UMIPIC follow-up and all-cause mortality, modeled with restricted cubic splines

When age was modeled using restricted cubic splines, the interaction with UMIPIC was not statistically significant (*p* for interaction = 0.134). Nonetheless, the point estimates suggested a trend toward greater benefit of UMIPIC in older patients. These findings indicate that the age-related modification of its effect is more likely linear than nonlinear. (Fig. 2).

#### Heart failure readmissions

**Fig. 3:**
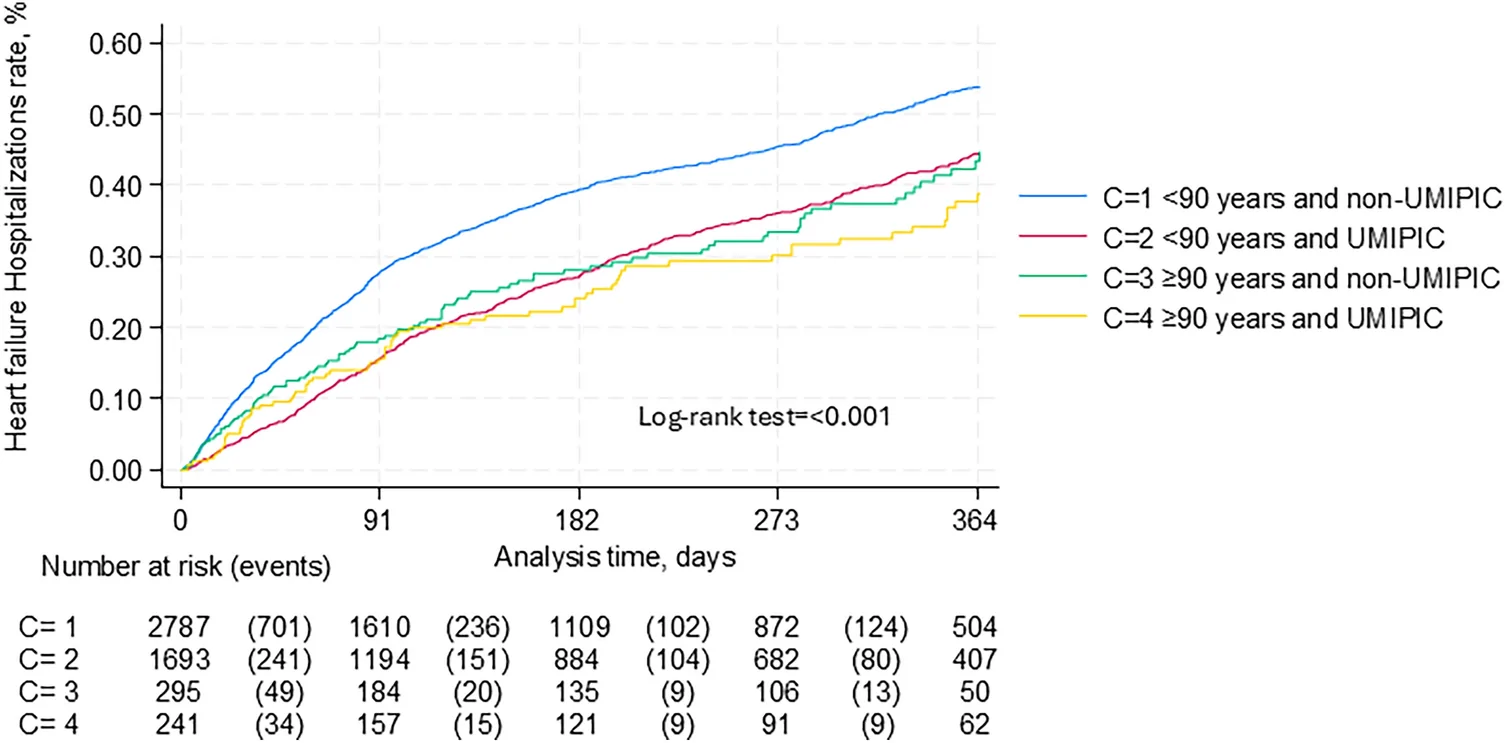
Kaplan-Meier curves age/UMIPIC categories for heart failure hospitalization

During the same follow up time, 1287 (22.8%) patients had heart failure hospitalizations (HFH). Patients with ≥ 90 years presented lower rates of HFH compared with < 90 years (19.1% vs 23.2%, p = 0.023). The predictive value of the care model (UMIPIC vs. non-UMIPIC), when stratified by age, revealed differential patterns of risk reclassification (C1 25.5%, C2 19.1%, C3 20.1%, C4 17.9%; *p* < 0.001). The Kaplan–Meier curves highlighted a pronounced excess risk in patients younger than 90 years without UMIPIC, while survival probabilities across the other strata did not differ substantially (Fig. 3). After multivariable adjustment, no significant interaction was observed between the care model (UMIPIC vs. non-UMIPIC) and age group on outcomes (*p* for interaction = 0.971). Using C = 2 as the reference (lower risk), patients in C = 1 showed a significantly higher risk of readmission (HR 1.56, 95% CI 1.36–1.78; *p* < 0.001). By contrast, C = 3 (HR 1.29, 95% CI 0.98–1.70; *p* = 0.072) and C = 4 (HR 0.83, 95% CI 0.58–1.19; *p* = 0.319) did not differ significantly from C = 2. When age was modeled as a continuous variable, the UMIPIC–age interaction indicated a trend toward stronger protection with advancing age (interaction HR 0.99 per year, 95% CI 0.97–1.00; *p* = 0.079), though this did not meet the threshold for statistical significance.

**Fig. 4:**
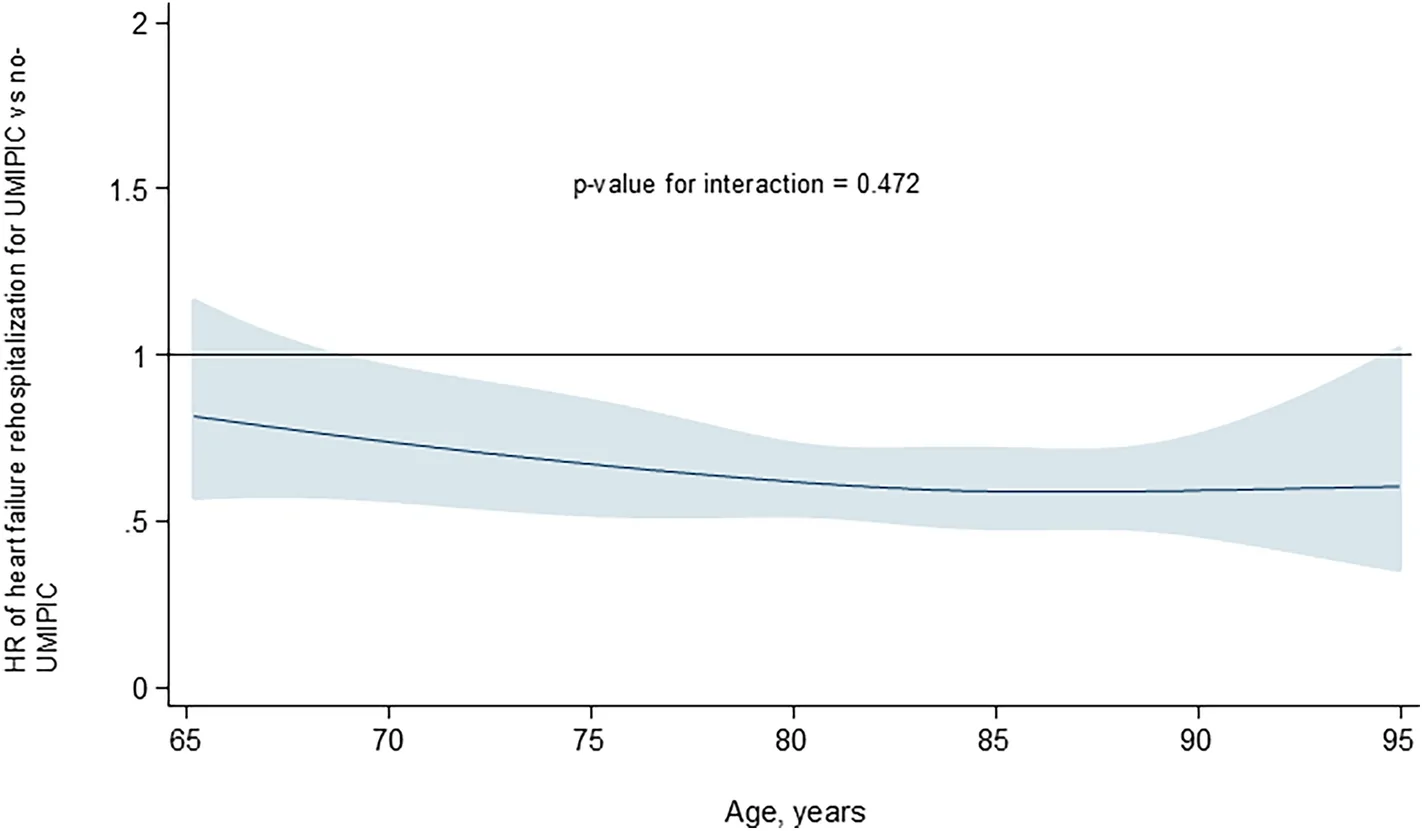
Effect of age on the association between UMIPIC follow-up and heart failure hospitalization, modeled with restricted cubic splines

Figure 4 shows the effect of the care model (UMIPIC vs. non-UMIPIC) on heart failure readmissions, modeled with a restricted cubic spline across the age spectrum. No significant interaction was observed (*p* for interaction = 0.472), indicating that the protective effect of UMIPIC was consistent across all ages.

## Discussion

In this large, prospective registry of older adults hospitalized for AHF, we observed that follow-up within the UMIPIC multidisciplinary program was consistently associated with improved outcomes, though the magnitude of benefit differed depending on the endpoint and age. Specifically, UMIPIC was associated with a substantial reduction in all-cause mortality, with evidence that this protective effect was even greater among the very elderly (≥ 90 years). In contrast, the benefit of UMIPIC in preventing rehospitalizations was robust and consistent across the entire age spectrum, without evidence of effect modification by age.

### Age, prognosis, and multidisciplinary care

Our findings align with prior evidence showing that age is a powerful determinant of mortality in patients with HF [13, 14] but also highlight that advanced age does not attenuate the benefits of structured multidisciplinary care. In fact, the significant UMIPIC-age interaction for mortality indicates that the survival advantage conferred by UMIPIC was amplified in the oldest patients. This may reflect the holistic, internist-led approach of the UMIPIC model [12, 15], which specifically addresses the high burden of multimorbidity, frailty, and functional vulnerability typical of nonagenarians [16–18].

Interestingly, nonagenarians in our cohort showed a paradoxical profile with fewer chronic comorbidities (lower Charlson index) but worse functional and cognitive status. This “survivor” phenotype, characterized by preserved organ health but greater frailty, may help explain their enhanced survival benefit with UMIPIC follow-up, which targets functional decline, early decompensation, and coordinated care. These findings suggest that biological age and functional reserve may better guide HF management in the oldest old than comorbidity burden alone.

Available evidence indicates that multidisciplinary heart failure programs benefit patients across all ages, although most studies and meta-analyses have been conducted predominantly in older adults. In clinical trials and systematic reviews, the mean age of participants usually ranges between 67 and 80 years, and the observed benefits include reduced mortality, fewer rehospitalizations, and improved treatment adherence, without identification of a specific age subgroup that benefits disproportionately compared to others [19–21].

Subgroup analyses in recent studies, such as that by Kamiya et al. [22], show that participation in multidisciplinary programs, including cardiac rehabilitation, is associated with significant prognostic benefits in heart failure regardless of age, sex, frailty, or the presence of comorbidities. Specifically, elderly and frail patients also experience reductions in mortality and rehospitalizations, with no evidence that benefits are attenuated in these groups.

Although UMIPIC is not a formal cardiac rehabilitation program with structured supervised exercise sessions, individualized physical activity counseling is systematically incorporated and tailored to functional status and frailty, with referral to dedicated rehabilitation programs when appropriate. In addition, given the advanced age and vulnerability of many participants, goals-of-care discussions and advance care planning are routinely integrated into follow-up through shared decision-making with patients and caregivers. These components may contribute to the observed survival benefit in the very elderly, beyond conventional pharmacological optimization.

Expert consensuses recommend a multidisciplinary approach for all patients with heart failure, emphasizing treatment individualization in older adults given the higher prevalence of comorbidities, polypharmacy, and frailty, but without restricting benefit to any specific age group [23, 24].

Our results provide evidence that even the most elderly patients, often underrepresented in clinical trials, derive substantial survival benefit from structured follow-up.

### Divergence between mortality and rehospitalization outcomes

Interestingly, age did not modify the impact of UMIPIC on HF readmissions. The program reduced the risk of readmission by about one-third across all age groups, and age-program interaction tests were not significant whether age was modeled dichotomously, continuously, or with restricted cubic splines. This pattern aligns with prior evidence that multidisciplinary HF programs consistently lower hospitalization risk, regardless of subgroup, and are recommended in contemporary guidelines [10, 11]. By contrast, readmissions are often precipitated by factors such as volume overload, suboptimal self-care/adherence, and exacerbations of noncardiac comorbidities, mechanisms that multidisciplinary models are designed to address broadly rather than differentially by age. Mortality, however, appears more sensitive to age-related vulnerability (e.g., frailty and multimorbidity), which is strongly associated with worse survival in HF and may be mitigated more effectively by comprehensive, internist-led care like UMIPIC.

### Clinical implications

These findings have relevant clinical implications. First, they challenge the notion that very advanced ages should limit referral to multidisciplinary programs; on the contrary, nonagenarians appear to benefit most in terms of survival. Second, the consistent reduction in readmissions across ages reinforces the broad applicability of UMIPIC in routine practice. Given the high healthcare burden of recurrent hospitalizations, scaling up structured programs such as UMIPIC could have substantial system-wide benefits.

### Limitations

Several limitations should be acknowledged. Firstly, as an observational analysis, residual confounding cannot be excluded despite adjustment for multiple covariates. Secondly, enrollment into UMIPIC was not randomized but determined by hospital availability and clinical judgment, raising the possibility of selection bias. Thirdly, our data derive from internal medicine settings in Spain, which may affect generalizability to other healthcare systems.

## Conclusions

UMIPIC follow-up was independently associated with reduced mortality and rehospitalizations in older patients with heart failure. The survival benefit was particularly pronounced among patients ≥ 90 years, whereas the reduction in rehospitalizations was consistent across the age spectrum. These findings underscore the importance of offering multidisciplinary, internist-led heart failure programs to elderly patients, including the very old, who represent a growing and particularly vulnerable segment of the heart failure population.

## Data Availability

The data that support the findings of this study are derived from the Spanish RICA registry. Restrictions apply to the availability of these data, which were used under agreement for the current study and are not publicly available. Data may be available from the corresponding author upon reasonable request and subject to approval by the RICA registry steering committee.
